# Temporal Observation of Adipocyte Microfiber Using Anchoring Device

**DOI:** 10.3390/mi10060358

**Published:** 2019-05-29

**Authors:** Akiyo Yokomizo, Yuya Morimoto, Keigo Nishimura, Shoji Takeuchi

**Affiliations:** 1 Center for International Research on Integrative Biomedical Systems (CIBiS), Institute of Industrial Science (IIS), The University of Tokyo, 4-6-1 Komaba, Meguro-ku, Tokyo 153-8505, Japan; yokomizo@iis.u-tokyo.ac.jp (A.Y.); y-morimo@hybrid.t.u-tokyo.ac.jp (Y.M.); nishimura@hybrid.t.u-tokyo.ac.jp (K.N.); 2 Department of Mechano-Informatics, Graduate School of Information Science and Technology, The University of Tokyo, 7-3-1 Hongo, Bunkyo-ku, Tokyo 113-8656, Japan; 3 Department of Life Sciences, Graduate School of Arts and Sciences, The University of Tokyo, 7-3-1 Hongo, Bunkyo-ku, Tokyo 113-8656, Japan; 4 International Research Center for Neurointelligence (WPI-IRCN), The University of Tokyo Institutes for Advanced Study (UTIAS), The University of Tokyo, 7-3-1 Hongo, Bunkyo-ku, Tokyo 113-8656, Japan

**Keywords:** microfluidics, biofabrication, adipose tissue, lipolysis

## Abstract

In this paper, we propose an anchoring device with pillars to immobilize an adipocyte microfiber that has a fiber-shaped adipocyte tissue covered by an alginate gel shell. Because the device enabled the immobilization of the microfiber in a culture dish even after its transportation and the exchange of the culture medium, we can easily track the specific positions of the microfiber for a long period. Owing to the characteristics of the anchoring device, we successfully performed temporal observations of the microfiber on the device for a month to investigate the function and morphology of three-dimensional cultured adipocytes. Furthermore, to demonstrate the applicability of the anchoring device to drug testing, we evaluated the lipolysis of the microfiber’s adipocytes by applying reagents with an anti-obesity effect. Therefore, we believe that the anchoring device with the microfiber will be a useful tool for temporal biochemical analyses.

## 1. Introduction

Core-shell cell microfibers, a fiber-shaped cellular-tissue covered by a shell of alginate gel, have become attractive in various applications, such as tissue engineering, cell therapy, and drug testing [1], because the three-dimensional (3D) culture of cells are performed at the core, and the alginate gel shell protects the cells from physical stimuli while allowing the diffusion of nutrition and oxygen [2]. Hence, core–shell cell microfibers have been widely used to construct various types of tissues including connective [2,3,4,5], neural [6], stem cell [7,8,9,10], smooth muscle [11], and adipose tissues [12]. Particularly, the culture of adipocytes in microfiber is a promising approach in the construction of adipose tissue, as the microfiber maintains the 3D culture of adipocytes by covering with the shell of the alginate gel for a long period. Therefore, core–shell adipocyte microfibers achieved the formation of large lipid droplets comparable to living adipose tissues. The adipocyte microfiber has advantages in culture dimension, handleability, and high-throughput production [12], compared to the recent methods for the adipocyte tissue formation with synthetic scaffolds and structures of extracellular matrix [13]. Moreover, conventional two-dimensional (2D) culture methods induce the detachment of adipocytes from a culture dish because of the increase of the adipocyte buoyancy during lipid accumulation [13,14]. Meanwhile, although the 2D culture enables the studies of lipid metabolism [15,16,17], the adipocyte microfibers cannot investigate the time course of changes in the function and morphology of the adipocytes because the microfibers move freely in a culture medium during their transportation and the exchange of medium.

**Figure 1:**
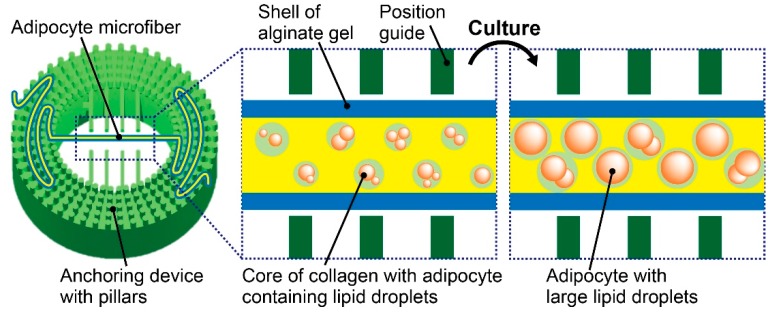
Conceptual illustration for immobilization of an adipocyte microfiber using the proposed anchoring device. By tangling the microfiber with pillars on the device manually, the microfiber becomes observable at the hollow section of the device for a long term without damaging the adipocytes.

In this paper, we propose a donut-shaped anchoring device with pillars to tangle an adipocyte microfiber (Figure 1). By placing the microfiber at a hollow section in the device and immobilizing both ends of the microfiber at the pillars, the microfiber can be observed clearly for a long period without damaging the cells in the microfiber. In addition, by referring to the position guides of the anchoring device, tracking a specific site in the microfiber is possible even after placing it out of the field of view of the microscope. Here, to evaluate the characteristics of the anchoring device, we set the adipocyte microfibers on the device and investigate the effect on the maturation of the adipocytes. Furthermore, by long periods of clear observations, we demonstrate that the anchoring device facilitates in the continuous observation of cellular morphology in the microfiber and evaluation of fatty acid release from the adipocyte microfiber by applying reagents, as an example of a drug testing application.

## 2. Materials and Methods 

### 2.1. Cell Preparation

3T3-L1 cells (mouse adipocytes, JCRB Cell Bank, Osaka, Japan, Cell No. JCRB9014) were seeded and maintained according to the manufacturer’s instructions, using a growth medium that was Dulbecco’s modified eagle medium low glucose (DMEM LG, 041-29775, FUJIFILM Wako Pure Chemical Corp., Osaka, Japan) containing 10% (v:v) fetal bovine serum (FBS (Chile Origin, USDA approved), FB-1365/500, Biosera, Nuaille, France), and 1% (v:v) penicillin/streptomycin (P/S, Sigma-Aldrich, St. Louis, MO, USA) at 37 °C in a 5% CO_2_ atmosphere. Passages were performed before the confluence of the cells.

### 2.2. Fabrication of the Anchoring Device

**Figure 2:**
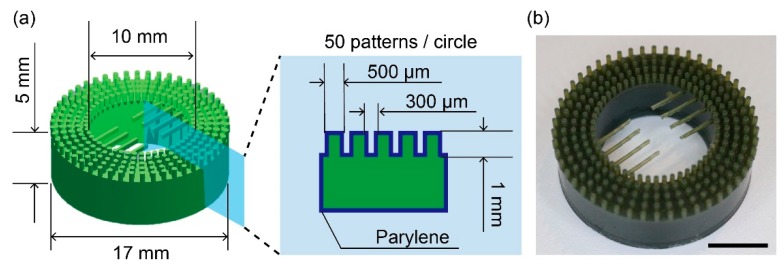
Design of the anchoring device with pillars: (**a**) Schematic illustration of the anchoring device with dimensions; (**b**) Image of the fabricated anchoring device. Scale bar is 5 mm.

The anchoring device for the immobilization of an adipocyte microfiber is 17 mm in outer diameter, 10 mm in inner diameter, and 6 mm in height (Figure 2); it is designed to be placed within a 35-mm culture dish and not to be floated in 2 mL of culture medium. Furthermore, 50 patterns of 5 pillars (500 µm in diameter, 1 mm in height) with 300-µm intervals were placed on the top surface of the device. The intervals were almost the same as the diameter of the microfiber. Bars of 0.3 mm width were arranged at the center of the device and used as a position guide for the identification of the observation area. The anchoring device was fabricated using a 3D printer (Perfactory 4 mini, Envision TEC, Dearborn, MI, USA). The fabricated device was coated with a 2-μm parylene layer using a chemical vapor deposition machine (Parylene Deposition System 2010, Specialty Coating Systems, Inc., Indianapolis, IN, USA) to improve the cell compatibility of the device [18,19].

### 2.3. Formation of Adipocyte Microfibers 

We fabricated adipocyte-laden hydrogel microfibers using a previously proposed method with a triple coaxial microfluidic device [2]. In the formation of the microfibers, a core solution, shell solution, and sheath solution were infused into the innermost channel, intermediate channel, and outermost channel of the microfluidic device, respectively. The flow rate of the core solution, i.e., a collagen solution (I-AC 50, KOKEN Co., Ltd., Tokyo, Japan) with 3T3-L1 cells at 1.0 × 10^8^ cells/mL, was 50 to 150 µL/min and that of the shell solution, i.e., a 1.5 wt% sodium alginate solution (194-13321, FUJIFILM Wako Pure Chemical Corp., Osaka, Japan), was 300 µL/min. The flow rate of the sheath solution, i.e., a 100-mM calcium chloride solution (191-01665, FUJIFILM Wako Pure Chemical Corp., Osaka, Japan) for gelling sodium alginate, was 3600 µL/min. After infusing the solutions into the microfluidic device, an adipocyte-laden hydrogel microfiber was formed; the fiber comprises an inner fiber-shaped adipocyte-laden collagen gel covered by an alginate gel layer. Subsequently, we placed the fabricated microfiber in the growth medium. After 2 days of culture, the growth medium was replaced with a differentiation medium (Preadipocyte Growth Medium-2 BulletKit™, PT-8002, Lonza, Basel, Switzerland). After 2 days of the culture with the differentiation medium, the medium was changed to a maturation medium that was DMEM high glucose (D5796, Sigma-Aldrich, St. Louis, MO, USA) containing 10% (v:v) FBS, 1% (v:v) P/S, and 5 µg/mL insulin (10516, Sigma-Aldrich, St. Louis, MO, USA). The maturation medium was replaced with a fresh medium every three days. Finally, the adipocytes were in contact with each other in the microfiber, thus resulting in the formation of adipocyte microfibers composed of a fiber-shaped adipocyte tissue covered by an alginate gel layer.

### 2.4. Microfiber Immobilization

**Figure 3:**
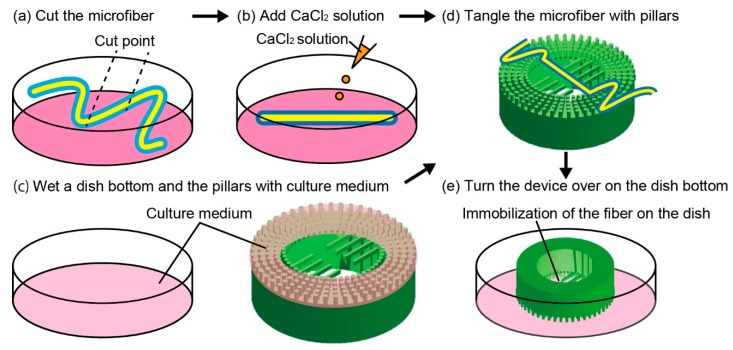
Process flow of immobilization of an adipocyte microfiber with an anchoring device: (**a**) Cut the microfiber with scissors; (**b**) Add CaCl_2_ solution to increase the strength of the alginate gel layer; (**c**) Wet a dish bottom and the pillars with a culture medium; (**d**) Tangle the microfiber with pillars using a pair of tweezers; (**e**) Turn the device over on the dish bottom for a clear observation.

For the immobilization of the adipocyte microfiber with the anchoring device, we first cut the microfiber to a length of ~5 cm with a pair of scissors such that the microfiber is mountable to the device (Figure 3a). To increase the strength of the alginate gel layer of the microfiber, we transferred it to a new dish containing a 1 mL aliquot of a 100-mM calcium chloride solution and 10-mL maturation medium (Figure 3b). Next, as a pretreatment of immobilization, we wetted a 35-mm dish containing the device with the culture medium to prevent the microfiber from adhering to the bottom of the dish; we also wetted the pillars on the device (Figure 3c). In addition, we used a static electricity removal gun and stopped the fan of the clean bench to prevent the microfiber from adhering to unintended positions. Subsequently, the microfiber was manually tangled with pillars using a pair of tweezers (Figure 3d). In this state, the surface tension of the culture medium between the pillars pushed the microfiber to the top surface of the device. Finally, to facilitate an observation with a microscope, we turned over the device on the dish bottom (Figure 3e). Then, the culture medium was injected gently from the outside of the device for the culture.

**Figure 4:**
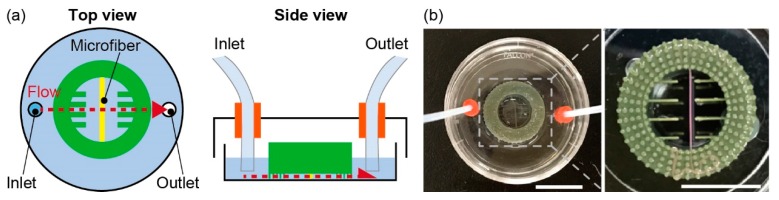
Experimental setup to evaluate immobilization of the microfiber in the anchoring device: (**a**) Conceptual illustration of applying flow to the microfiber in the device; (**b**) Image of the experimental setup. Scale bars are 10 mm.

To verify the immobility of the microfiber on the anchoring device during culture, we observed the movements of a fluorescent alginate hydrogel microfiber in the presence of disturbances. The fluorescent alginate hydrogel microfiber was fabricated by infusing a 1.5 wt% sodium alginate solution containing 1 µL/mL red fluorescent beads (F8810, Life Technologies Corp., Carlsbad, CA, USA) as a core solution and a 1.5 wt% sodium alginate solution with 1 µL/mL green fluorescent beads (F8811, Life Technology, Inc.) as a shell solution into the microfluidic device. When we applied a flow to the device as a disturbance, we connected syringes to the dish using septums and Teflon tubes (Figure 4). First, a 5-mm-thick silicone rubber sponge (5-3030-04, AS ONE, Osaka, Japan) was punched out with a trepan for biopsy, and 2 pieces of 4-mm-diameter rubber sponges with a 1-mm-diameter hole at the center were prepared. Subsequently, we pressed them into 2.5-mm diameter holes that were made on the cover of a 35-mm culture dish. Finally, we inserted the tubes into the septums and connected them to the syringe through silicone tubes. Then, we flowed sterilized water at 0.01 to 10 mL/min for 60 s and measured the distances between the guides of the device and the microfiber using a microscope (IX71N, Olympus Corp., Tokyo, Japan) and imaging software (Image J, 1.46r software package, National Institutes of Health (NIH), Bethesda, MD, USA). The distances were labeled as d_1_, d_2_, d_3_, and d_4_ in order from the left side of the images. In the case of applying rotations to the device, the dish with the device was placed on a turntable (NA-301, Nissinrika, Tokyo, Japan) and rotated at 30 to 120 rpm for 30 s. After the rotation, we measured the distances between the guides and microfibers using the same method as the measurement under an applied flow.

### 2.5. Morphology Evaluation of the Microfiber

To evaluate the dimensions of the adipocyte-laden hydrogel microfibers according to the flow rates of the infused flows in the microfluidic device, we prepared the microfibers with different flow rates of core solution (50 µL/min, 100 µL/min, 150 µL/min), and measured the diameters of the shell and core of the microfibers using a microscope (IX71N, Olympus Corp., Tokyo, Japan) and an imaging software (cellSens, Olympus Corp, Tokyo, Japan). Moreover, we measured the sizes of lipid droplets in the adipocytes of the cultured adipocyte microfibers using bright-field images or fluorescent images in the case of staining the lipid droplets with BODIPY 493/503 (D3922, Thermo Fisher Scientific, Waltham, MA, USA). In the measurement, we defined the diameter of the lipid droplets as the average value of the orthogonal diameters measured on an imaging software (Image J 1.46r software package, National Institutes of Health (NIH), Bethesda, MD, USA). To compare the lipid droplet size between the adipocyte microfibers and the 2D cultured adipocytes, we seeded the adipocytes to a dish at 18,000 cells/cm^2^ and changed the growth medium to the differentiation medium after confluence. After the adipocytes were cultured on a dish, we measured the diameters of the lipid droplets in the adipocytes using the same method as that for the adipocyte fibers.

To investigate the effect of the anchoring device to the culture of the adipocyte microfibers, we measured the size of the lipid droplets in the microfibers cultured on the device using the method above. In addition, we set an adipocyte microfiber on the anchoring device to observe the edge of an adipocyte tissue and verified the morphological changes in lipid droplets of the tissue. In the experiment, we used the maturation medium containing oleic acid-BSA complex (O3008, Sigma-Aldrich, St. Louis, MO, USA) at 500 µM. Five minutes after culture in the culture medium, we started the observation for the lipid droplets using an all-in-one microscope (BZ 9000, KEYENCE Corp., Osaka, Japan) and captured their images hourly for 24 h.

### 2.6. Evaluation of Lipolysis in Adipocyte Microfiber

To demonstrate the applicability of the adipocyte microfiber immobilized with the anchoring device to drug testing, we evaluated the lipolysis of the adipocytes in the microfiber. In the experiment, we first cultured the microfiber with 2 mL of a DMEM high glucose solution to prevent the effects of serum and insulin and 1 µL of 1 mg/mL BODIPY to dye the lipid droplets. After incubation for 3 h or more, we changed the culture medium to DMEM low glucose solution containing 2% (v:v) BSA (A7906, Sigma-Aldrich) and 5% (v:v) FBS. 5 min after the medium change, we started the observation of the microfiber using the all-in-one microscope and captured fluorescence images hourly for 6 h. Subsequently, we changed the culture medium to DMEM low glucose solution containing 2% (v:v) BSA, 5% (v:v) FBS, and 1 mM isoproterenol (I5627, Sigma-Aldrich, St. Louis, MO, USA). After 5 min of culture, we recorded fluorescence images hourly for 6 h. To get multiple data, we repeated the experiment after 24 h and more from the previous experiment. After repeating experiments three times, we extracted green-channel images from the captured full-color images and measured the fluorescence intensity of the microfiber using Image J. 

## 3. Results and Discussion

### 3.1. Characterization of the Anchoring Device

**Figure 5:**
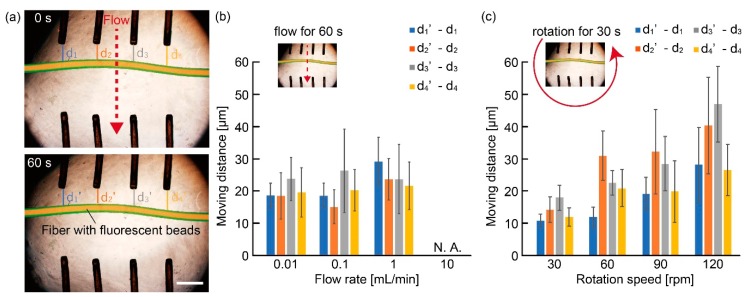
Evaluation of immobilization of a microfiber with fluorescent beads with the anchoring device: (**a**) Images of the microfiber before and after applying a 1 mL/min flow in the device; (**b**,**c**) Moving distance of the microfiber in the anchoring device when we applied (**b**) a flow and (**c**) a rotation to the device (N = 5, mean ± s. d.). Scale bar is 1 mm.

To verify the characteristics of the anchoring device for the immobilization of the adipocyte microfiber, we applied flows to the device with the fluorescent hydrogel fiber (Figure 5a). Consequently, we confirmed that the moving distance of the hydrogel microfiber induced by the flows was within 50 µm under a flow rate lower than 1 mL/min (Figure 5b). Meanwhile, when the flow rate was 10 mL/min, the hydrogel microfiber was immobilized on the device. However, the device moved, thus causing the microfiber to be outside the microscope’s field of view. In the case of applying rotations to the device, the moving distance was within 100 µm even when the rotation speed was 120 rpm (Figure 5c). These results indicate that our anchoring device can maintain the position of the microfiber even under disturbances, such as transportation of the culture dish and exchanges in the culture medium. Therefore, the anchoring device allows us to trace the specific position of the adipocyte microfiber during culture. Although immobilization has been achieved by reeling and weaving the microfibers and sucking both ends of the microfibers [2,3,4,10,20,21,22,23,24,25], both methods are not suitable for investigating the time course of cellular changes in the microfibers. The reeling and weaving cause difficulties in maintaining a clear observation because the microfibers may overlap each other. While sucking can immobilize the microfibers to avoid overlapping, it is not suitable for the investigation during culture because a culture medium is also sucked during the immobilization, resulting in dynamic changes in culture conditions. Thus, we believe that the anchoring device is a useful tool for the investigation of the microfibers by temporal observation.

### 3.2. Characterization of Adipocyte Microfiber

**Figure 6:**
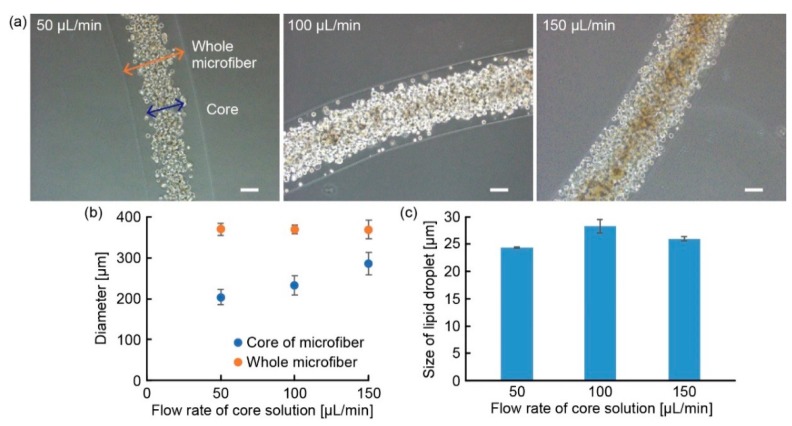
Relationship between flow rate of core solution and size of lipid droplets in adipocytes: (**a**) Images of adipocyte-laden hydrogel microfibers (the flow rate of core solution was 50 µL/min, 100 µL/min, 150 µL/min); (**b**) Relationship between diameters of the microfibers and flow rates of core solution under 300 µL/min flow of shell solution (N ≥ 5, mean ± s. e. m.); (**c**) Size of lipid droplets after 21 days of culture depending on the flow rate of the core solution (N = 3, mean ± s. e. m.). Scale bars are 100 μm.

To investigate the appropriate fabrication conditions for the formation of the adipocyte microfiber, we prepared the adipocyte-laden hydrogel microfibers under different flow rates of the core solution. Although the diameter of the whole fiber (the diameter of the alginate gel shell) was not changed significantly with the increase in the flow rate of the core solution, the diameter of the adipocyte-laden collagen core was increased (Figure 6a,b). This result indicates that it is possible to control the thicknesses of the shell and core without changing the diameter of the whole fiber by controlling the flow rate of the core solution, to induce changes in the culture condition for adipocytes under maintenance of mountable microfiber diameter (~ 300 μm) to the anchoring device. As a result of culturing for the maturation of adipocytes, we recognized that the adipocyte microfiber fabricated at 100 µL/min of the core solution exhibited larger lipid droplets than those fabricated at 50 and 150 µL/min of the core solution (Figure 6c). Therefore, in this paper, we decided to use the adipocyte microfiber prepared with 100 µL/min of the core solution in the following experiments.

**Figure 7:**
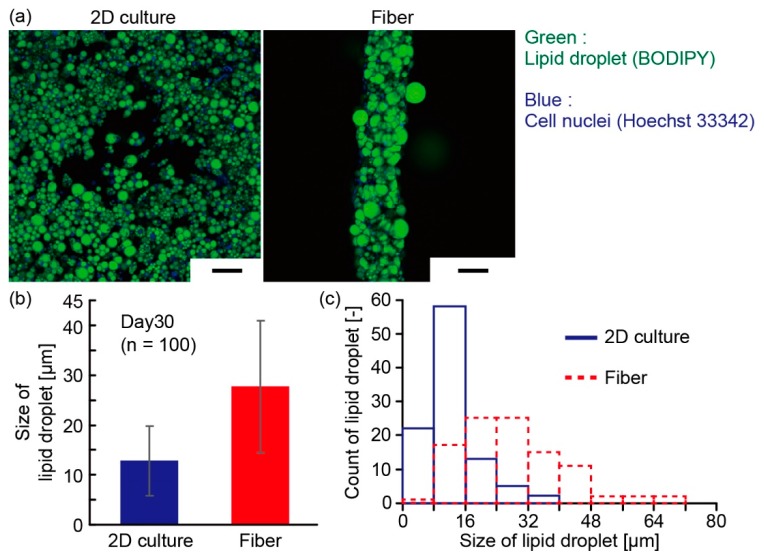
Comparison of 2D-cultured adipocytes in a dish and adipocytes in a microfiber: (**a**) Fluorescence images of lipid droplets in 2D culture and microfiber culture; (**b**) Average size (n = 100, mean ± s. d.) and (**c**) size distribution of lipid droplets on day 30 in both culture conditions. Scale bars are 100 μm.

To evaluate the effects of the culture using microfiber for the adipocytes, we compared the morphology of the adipocytes cultured in the microfiber with those cultured in a 2D culture dish. While the adipocytes in 2D culture became sparse, the adipocytes in the microfiber became dense adipose tissue as in vivo (Figure 7a). The average lipid droplet size of the microfiber was 28 ± 13 µm, which was approximately twice larger than that of the 2D culture, 13 ± 7 µm (mean ± s. d.) (Figure 7b). In addition, the peak of the size distribution of the lipid droplets in 2D culture was as small as 10 µm, while the size of lipid droplets in the microfiber was distributed widely from 10 µm to 50 µm (Figure 7c). The formation of large lipid droplets in the adipocytes is important to mimic the state of obesity because the responses of adipocytes to biochemicals change depending on the size of the lipid droplets [26,27]. Therefore, these results indicate that the adipocyte microfiber provides an adipocyte tissue which is more useful in the investigation of biochemical reactions under obesity than 2D cultured adipocytes. 

**Figure 8:**
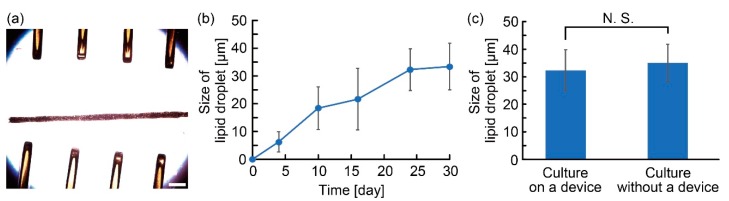
Observation of an adipocyte microfiber cultured on the anchoring device: (**a**) Images of an adipocyte microfiber on day 28; (**b**) Size of lipid droplets varying with time (n ≥ 10, mean ± s. d.); (**c**) Comparison of lipid droplet size in adipocyte microfibers cultured with or without the anchoring device for 24 days (n = 20, mean ± s. d.). Scale bar is 500 μm.

**Figure 9:**
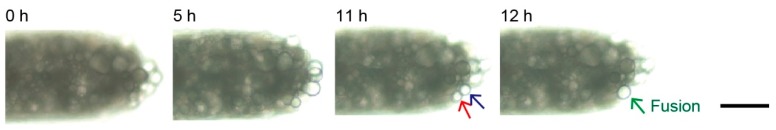
Time-lapse images of an adipocyte microfiber on the anchoring device. A fusion (green arrow) of lipid droplets (red and blue arrows) was observed. Scale bar is 100 μm.

Furthermore, we investigated the culture characteristics of the adipocyte microfiber on the anchoring device. By tangling the microfiber with pillars on the device, we achieved the immobilization of the adipocyte microfiber and clear observation of the adipocytes (Figure 8a). Owing to the features of the device, we successfully evaluated the time-dependent change of the size of the lipid droplets for a month (Figure 8b). In the comparison of the lipid droplet sizes when cultured with or without the device, no significant difference was indicated (*p* > 0.2, Student’s *t*-test) (Figure 8c). This result indicates that the anchoring device does not affect the maturation of the adipocytes in the microfiber. Moreover, we continuously observed the edge of an adipocyte tissue in an adipocyte microfiber. Consequently, we confirmed the migration of lipid droplets, changes in their size, and fusion of the droplets in the adipocyte microfiber (Figure 9). This result indicates that the anchoring device allows us to observe the behaviors of single adipocytes in the microfiber, leading to studies of lipid metabolism based on the morphology of the lipid droplets.

### 3.3. Lipolysis of Adipocyte Microfiber

**Figure 10:**
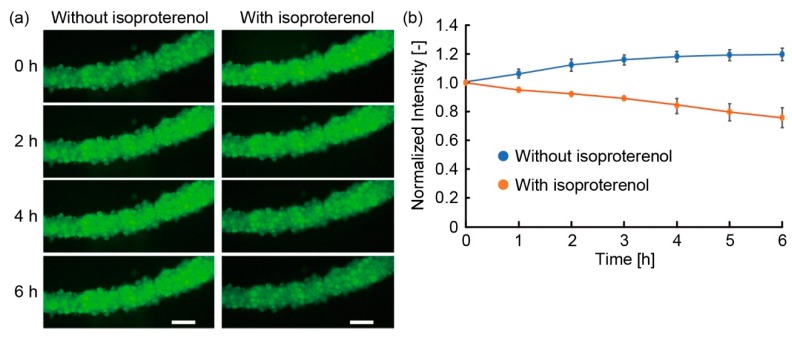
Changes in fluorescence intensity of lipid droplets stained with BODIPY when applying isoproterenol to the adipocyte microfiber on the anchoring device: (**a**) Time-lapse fluorescent images of the microfiber on the anchoring device; (**b**) Variation with time of fluorescence intensity of the adipocyte microfiber in specific observation area under repeating the experiment three times (mean ± s. d.). The fluorescent intensity is normalized with that at 0 h. Scale bars are 100 μm.

To demonstrate the applicability of the adipocyte microfiber immobilized with the anchoring device to drug testing, we examined the lipolysis of the adipocyte microfiber using isoproterenol, a reagent with anti-obesity effect. When we added isoproterenol to the microfiber stained with BODIPY for the visualization of lipids on the device, the fluorescence intensity of the microfiber decreased as time progressed, in contrast to the fluorescence intensity of the microfiber without the addition of isoproterenol (Figure 10). As the exposure times were the same in both experiments, the decrease in fluorescence intensity was not caused by the quenching of BODIPY with laser irradiation. Therefore, the results indicate that the decrease was caused by the release of fatty acids from the adipocytes, demonstrating that lipolysis by isoproterenol was achieved in the microfiber. Hence, the anchoring device enabled the observation of lipolysis by the immobilization of the microfiber. Therefore, the feature of the device enabling the temporal observation of the specific position in the microfiber is useful in lipid metabolism research or early-stage drug screening.

## 4. Conclusions

In this study, we developed an anchoring device with pillars for the temporal observation of an adipocyte microfiber. The advantages of the anchoring devices are as follows: (i) temporal observation of the specific position in the microfiber even when applying disturbances; (ii) maturation of adipocytes in the microfiber on the anchoring device comparing to that without the device; (iii) clear observation of lipid droplets and cell morphology in the microfiber. Based on these advantages, we show that the adipocyte microfiber immobilized on the anchoring device could be used for drug testing by enabling the study of lipolysis under the addition of isoproterenol. Although we focused on the adipocyte microfiber herein, the anchoring device enabled the temporal observation of various types of cell microfibers. Therefore, we believe that the anchoring device will be a useful tool for studies in various fields, such as biochemistry and drug testing.
